# Associations Between Adherence to the EAT-Lancet Planetary Health Diet and Nutritional Adequacy, and Sociodemographic Factors Among Australian Adults

**DOI:** 10.3390/nu18020340

**Published:** 2026-01-21

**Authors:** Jayden B. Ordner, Claire Margerison, Linda A. Atkins, Ewa A. Szymlek-Gay

**Affiliations:** Institute for Physical Activity and Nutrition (IPAN), School of Exercise and Nutrition Sciences, Deakin University, Melbourne Burwood Campus, 221 Burwood Highway, Burwood, VIC 3125, Australia; claire.margerison@deakin.edu.au (C.M.); l.atkins@deakin.edu.au (L.A.A.); ewa.szymlekgay@deakin.edu.au (E.A.S.-G.)

**Keywords:** 2025 EAT-Lancet Commission, sustainable food systems, dietary assessment, dietary patterns, nutritional epidemiology, food sustainability, Australian diets

## Abstract

**Background/Objectives:** Adherence to the EAT-Lancet Planetary Health Diet (PHD) may promote human health and environmental sustainability, yet evidence regarding adherence and nutritional adequacy in Australia is limited. Globally, no research to date has used the recently updated 2025 PHD guidelines. We benchmarked the compatibility of Australian adults’ dietary patterns with the 2025 PHD and examined its associations with nutritional adequacy and sociodemographic factors. **Methods:** This was a cross-sectional analysis of dietary data from 5655 adults who participated in the National Nutrition and Physical Activity Survey. Usual intakes were estimated from two 24 h recalls using the Multiple Source Method. PHD adherence was measured using the Healthy Reference Diet Score (0–130 points). Nutrient adequacy was assessed using the full probability method for iron and the Australian/New Zealand Estimated Average Requirement Cut-Point Method for all other nutrients. Survey-weighted regression models examined associations with nutritional adequacy and sociodemographic factors. **Results:** The mean PHD adherence score was 50 (SE 0.3) points. Higher adherence was associated with lower odds of inadequate intakes of several micronutrients, but with higher odds of inadequacy for vitamin B12 (OR: 1.24; 95% CI: 1.06, 1.45) and calcium (OR: 1.09; 95% CI: 1.01, 1.17). PHD adherence was higher among females, older adults, those with higher educational attainment, those born in countries where English is not the main language, two-person households and non-smokers; adherence was non-linearly associated with alcohol and was lower among those with a Body Mass Index ≥ 30 kg/m^2^. **Conclusions:** PHD adherence in Australia was low. Higher adherence was associated with improved adequacy for several micronutrients. Trade-offs for vitamin B12 and calcium warrant consideration. Equity-conscious strategies will be needed to support the adoption of nutritionally adequate, environmentally sustainable diets.

## 1. Introduction

The necessity of radical global food system transformation under the duress of climate change has exposed knowledge gaps in how to ensure both nutritional adequacy and environmental sustainability [1,2]. The ‘Planetary Health Diet’ (PHD), developed by the EAT-Lancet Commission on Food, Planet and Health in 2019 and updated in 2025, is a landmark framework for a healthy and environmentally sustainable plant-based diet [3,4]. It predominantly emphasises wholegrains, fruits, vegetables and legumes in favour of reducing the consumption of red meats and added sugars, while recommending moderate intakes of dairy, poultry and seafood. Adhering to the PHD is proposed to provide nutritional adequacy, reduce chronic disease burden and match nutrition recommendations with emerging sustainability and climate-related goals [4]. It is well established that population nutrition in economically developed nations cannot continue to be dominated by the overconsumption of environmentally intensive animal-source foods such as beef and dairy [5,6]. However, the degree of animal-source food restriction within the 2019 PHD recommendations has attracted criticism and debate, particularly regarding potential micronutrient shortfalls, most notably vitamin B12, iron, zinc and calcium [7]. Inadequate intakes of nutrients like these, commonly found in red meats, dairy and other animal-source foods, may increase the risk of deficiency and disease if not adequately substituted for a diversity of plant-based foods, including those with fortification [8]. The 2025 PHD report has since described calcium, iodine, iron and vitamin B12 as nutrients requiring further attention and emphasised that the PHD reference ranges are sufficiently flexible to support the optimisation of food intakes across diverse contexts to ensure nutritional adequacy for all population groups [4].

To date, no research has examined the nutritional implications of the 2025 PHD. Globally, studies examining associations between 2019 PHD adherence and nutritional adequacy have produced mixed results [9,10,11,12,13,14]. Some research suggests that higher 2019 PHD adherence decreases the risk of inadequacy for vitamins B6, B9 and C, as well as magnesium and zinc, but increases the risk of inadequacy for vitamin B12 and calcium [13]. Others report that high adherence reduces the probability of iron inadequacy [9], while elsewhere, overall nutrient adequacy improves; however, for some nutrients, there may be no difference between lower or higher adherence [11]. In Australia, evidence is limited. Australians reportedly exceed the 2019 PHD recommendations for the consumption of meat (by 258%) and dairy (by 161%), indicating low overall adherence to its guidelines [15]. However, it remains unclear whether greater adherence to the PHD would support nutritionally adequate diets in the Australian population. Given the various calls to action from nutrition organisations to consider sustainability during the development of dietary guidelines and nutrition policy, there is a pressing need to better understand how diets like the PHD intersect with population nutrition in Australia [16,17,18].

In addition to understanding nutritional adequacy, it is important to explore the broader factors that influence PHD adherence. Dietary behaviours are shaped by a range of sociodemographic factors such as age, sex, education and socioeconomic disadvantage [19]. Identifying these associations can help contextualise what healthy and sustainable diets look like in practice relative to actual dietary intake patterns and the diversity of the Australian population.

The objectives of this study were to (1) benchmark the compatibility of Australian adults’ dietary patterns with the 2025 PHD, (2) examine associations between PHD adherence and nutritional adequacy and (3) identify sociodemographic factors associated with PHD adherence.

## 2. Materials and Methods

### 2.1. Study Design and Participants

**Figure 1:**
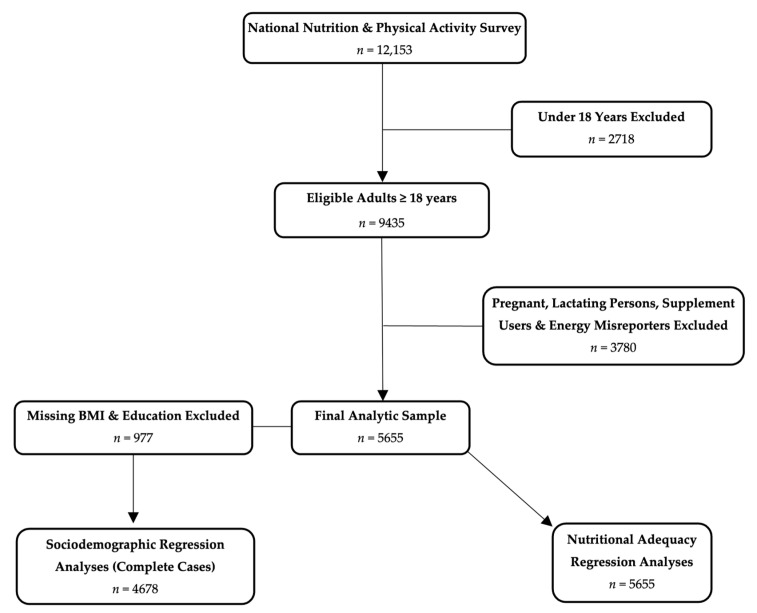
Flow chart of eligible and excluded participants screened for the present study.

This study involved a secondary analysis of the 2011–2012 National Nutrition and Physical Activity Survey (NNPAS) component of the Australian Health Survey, which has been described in detail elsewhere [20]. In brief, the Australian Health Survey was a cross-sectional, nationally representative study conducted by the Australian Bureau of Statistics using random multistage area sampling which identified 9500 dwellings from all Australian states and territories across both urban and rural areas. Household and person-level weights were assigned to account for both selection probability and non-response to enable population-representative estimates for Australia. Computer-assisted personal interviews with one responsible adult from each dwelling were used to determine the general characteristics of the household, and from this information, one adult aged ≥18 years was selected for inclusion. Data on socioeconomic status, demographic variables, anthropometric measures and dietary intake were recorded. The final NNPAS sample contained data from 12,153 participants aged ≥2 years, of whom 9435 were adults aged ≥18 years and were eligible for inclusion in the present study (Figure 1).

### 2.2. Sociodemographic Data

The sociodemographic data included in these analyses were age, sex (male/female), education level (did not complete secondary school [<Year 12], completed secondary school [Year 12], certificate/trade or diploma, bachelor’s degree, or postgraduate degree), country of birth (Australia, major English-speaking countries (United Kingdom [England, Scotland, Wales, Northern Ireland], Republic of Ireland, New Zealand, Canada, United States of America and South Africa) or other countries), household size (categorised as 1, 2, 3 or ≥4 people per household), Index of Relative Socio-economic Disadvantage (IRSD; this index ranked areas by socioeconomic disadvantage using 16 key socioeconomic indicators (some of which were % of households with low income, % of people without qualifications and % of people in low skilled occupations) divided into quintiles ranging from the most disadvantaged [lowest quintile] to the least disadvantaged [highest quintile]), labour force status (employed, unemployed or not in the labour force) and current smoking status (yes/no) [20].

### 2.3. Anthropometric Data

Participants’ height and weight were measured by trained interviewers using a portable stadiometer and digital scales [20]. BMI was calculated as kg/m^2^ and categorised as underweight (<18.5), healthy weight range (18.5 to <25), overweight (25 to <30) or obese (≥30) [20].

### 2.4. Dietary Data

Dietary data used in these analyses were collected in the NNPAS between 2011 and 2012. Participants completed two non-consecutive 24 h dietary recalls using the 5-step United States Department of Agriculture automated multiple-pass method adapted for the Australian food system [20]. The first recall was conducted during the in-person interviews, with the second conducted at least 8 days later via a computer-assisted telephone interview. All NNPAS participants provided at least one 24 h recall, with 3456 adults providing a second recall. Pamphlets containing depictions of foods, serving sizes and containers were supplied to assist participants with the recollection of meals and their portion sizes. The dietary data were coded (name, description, 8-digit code) and analysed (energy and nutrients) using the AUSNUT 2011–2013 food database, a collaboration between the Australian Bureau of Statistics and Food Standards Australia and New Zealand [20]. The database contained 5740 foods, 15,847 measures and food preparation methods (e.g., cooked, raw, fortified) [20].

### 2.5. Analytic Sample

NNPAS participants aged ≥18 years were eligible for inclusion in the current study (*n* = 9435). Pregnant and lactating individuals were excluded, as the PHD was not specifically developed to meet the nutritional needs of these individuals. Individuals who reported consuming any supplements were also excluded. Misreporters of energy intake were then identified and excluded using the method described by Huang et al., which compares reported energy intake to predicted total energy expenditure [21]. This was estimated using the Institute of Medicine’s sex and age-specific coefficients for predicting total energy expenditure [22]. The final analytic sample comprised 5655 adults (Figure 1).

### 2.6. Usual Food and Nutrient Intakes

The dietary data were used with the Multiple Source Method (Version 1.0.1; Department of Epidemiology, German Institute of Human Nutrition, Potsdam-Rehbrücke, Germany) to estimate the usual intakes of energy, fats (saturated and unsaturated), and all nutrients reported in the NNPAS that have an Australian/New Zealand Estimated Average Requirement [23,24]. This included vitamins A, B1, B2, B3, B6, B9, B12, C, D and E and the minerals calcium, iodine, iron, magnesium, phosphorus, selenium and zinc. For energy, fats and nutrients, the Multiple Source Method’s default assumption of habitual consumption was used in the models. Usual intake estimates of 12 dietary components (wholegrains, vegetables, fruits, legumes, starchy vegetables, dairy, eggs, poultry, fish and seafood, nuts and seeds, red meats, and added sugars) were also modelled using the Multiple Source Method for all participants. For individuals who did not report consumption of a dietary component on a recall day, a probability value of 0.5 was applied to reflect potential episodic consumption, allowing usual intake estimations for 50% of those individuals [23]. Usual alcohol consumption estimates were specified using a consumption probability of 0.81 based on the proportion of adults who reported alcohol consumption in the 2013 National Drug Strategy Household Survey [25]. All usual intake models were adjusted for age, sex, day type (weekday or weekend) and recall sequence (first or second).

### 2.7. Estimation of Planetary Health Diet Adherence

Usual intakes of dietary components and fats (g/day) were used to measure 2025 PHD adherence using an adaptation of the Healthy Reference Diet Score (HRDS) (Table 1) [26]. In brief, the HRDS uses each of the 13 dietary recommendations outlined in the PHD, which are organised into adequacy, optimum, moderation and ratio components. Individuals receive proportional scores for each dietary component, ranging from 0 to 10 points, based on how closely their usual intake aligns with the recommended intake targets. For an adequacy component, such as vegetables, the recommended intake is 300 g/day for those consuming 2400 kcal/day. Intakes are scored proportionally starting from 0 points at 0 g/day to 10 points at 300 g/day, with no modification to the score for exceeding this recommendation.

Optimum components combine a different proportional and inverse scoring design. For example, for eggs (based on 2400 kcal/day), scores proportionally scale from 0 to 10 points for intakes between 0 g and 15 g/day. Egg intakes between 15 and 25 g/day score 10 points, representing an optimum intake range. However, for intakes between 25 and 40 g/day, scores become inversely proportional, falling from 10 to 0 points. Intakes above 40 g/day score 0 points; these optimum cut-offs are based on the PHD’s reference values [4]. Moderation components are scored inversely, penalising higher-impact dietary components. For added sugars (based on 2400 kcal/day), scoring starts at 0 points for 31 g/day and proportionally increases to 10 points for 0 g/day. Due to a lack of disaggregated data for the added fats PHD category (palm oils, lard, tallow, ghee and butter), a ratio of total unsaturated to total saturated fats was used for the HRDS ratio component, consistent with the approach taken in previous research [26,28]. Cut-offs for this ratio component were derived from the 15th percentile (i.e., a ratio of 1.072) and the 85th percentile (i.e., a ratio of 1.829) of intake values observed in our sample, in line with methods described in earlier research [29]. Ratios of unsaturated to saturated fats at or below 1.072 scored 0 points, ratios at or above 1.829 scored 10 points, and intermediate values received proportional scores between these cut-offs. A total of 130 points represents the highest possible PHD adherence score according to this design. To account for variation in individual energy intake, we energy-adjusted the gram-based targets for each of the dietary components (adequacy, optimum and moderation) for each participant by scaling them to each participant’s usual energy intake. This was performed by dividing each participant’s usual energy intake by the PHD reference energy intake of 2400 kcal/day and multiplying each individual dietary component’s target intake by the resulting ratio; the unsaturated/saturated fat ratio component of the HRDS was excluded from this adjustment as it was derived from sample percentiles. The foods included in each dietary component are listed in Supplementary Table S1.

### 2.8. Adequacy of Nutrient Intakes

The Estimated Average Cut-Point Method was used to classify individuals as having either adequate or inadequate nutrient intakes based on age- and sex-specific Australian/New Zealand Estimated Average Requirement values [24]. This method is appropriate for nutrients where requirements are symmetrically distributed around the mean [30]. For iron, the full probability method was used, as the distribution of iron requirements, particularly among menstruating females, is not symmetrical around the mean [24,30].

### 2.9. Statistical Analysis

Descriptive statistics were used to summarise sociodemographic characteristics, mean PHD adherence scores, usual intakes (energy, nutrients and alcohol), and the HRDS dietary components, both for the whole sample and per quartile of PHD adherence; these variables were each assessed for normality. Survey-weighted means, standard errors, medians, interquartile ranges (25th and 75th percentile) and percentages were calculated, as appropriate, using the survey sampling weights to account for the complex survey design.

Logistic regression was used to assess the associations between PHD adherence scores and nutritional adequacy (adequate/inadequate). To facilitate interpretation, adherence scores were rescaled by a factor of 10 (from a range of 0–130 to 0–13), and logistic regression was used to estimate the odds of being at risk of inadequate intake per 10-point change in adherence score. Iron was excluded from this analysis due to the requirement for the full probability method. Instead, linear regression was used to assess the association between PHD adherence scores and the probability of inadequate iron intake, as estimated using the full probability method. These probabilities were modelled as a continuous outcome, and regression analysis was performed to estimate the change in probability per 10-point increase in adherence score. Nutritional adequacy models were also fitted using unscaled adherence scores (0–130), which reflected a 1-point increase in adherence. Potential non-linearity was examined for all nutrients using restricted cubic splines and Wald’s test of non-linear components. All nutritional adequacy models were adjusted for the covariates of age, sex and energy intake. Due to the very low number of individuals with inadequate intakes of vitamin B3 and phosphorus, regression modelling was not feasible.

Univariable linear regression was used to identify potential sociodemographic factors associated with PHD adherence scores, and each was adjusted for energy intake. Due to missing data for BMI (*n* = 913) and education (*n* = 77), these models were restricted to a complete case sample (*n* = 4678; Figure 1); multiple imputation was not implemented. Alcohol consumption, which showed a non-linear association with PHD adherence scores, was modelled using restricted cubic splines. Regression diagnostics were performed on all univariable regression models to assess model assumptions (linearity, homoscedasticity and the normality of residuals). Factors with *p*-values < 0.2 were subsequently included in a multivariable linear regression model to identify independent associations. Post-estimation multicollinearity was evaluated using variance inflation factors.

Jackknife survey weights were applied to account for the NNPAS design and enable inference to the Australian population. All proportions and estimates were calculated, and regression models were fitted using survey commands. Statistical significance was defined as *p* < 0.05. All analyses were conducted between May and October 2025 using STATA 18.0 (StataCorpLLC. College Station, TX, USA).

## 3. Results

### 3.1. Population Characteristics

The final analytic sample included 5655 adults (Figure 1) with a median (25th, 75th percentile) age of 43 (29, 57) years (Table 2). Approximately 60% of participants were living with overweight or obesity. Most participants were born in Australia (70%), and 11% were from major English-speaking countries. Over half of the participants (58%) had completed a post-secondary school education. The sample was socioeconomically diverse, with approximately equal representation across IRSD quintiles. Nearly one-third (32%) of participants lived in two-person households, and 36% lived in households with four or more people. Most participants were employed (68%), 21% currently smoked and the median (25th, 75th percentile) usual alcohol consumption was 3.7 g/day (0.9, 19.9) (Table 2).

### 3.2. Usual Dietary Intakes

Usual dietary component intakes are reported in Table 3. The lowest scoring dietary components (median [25th, 75th percentile]) were legumes (0.6 [0.0, 2.0] points), nuts and seeds (0.6 [0.0, 1.7] points), wholegrains (0.4 [0.0, 1.4] points), added sugars (0.0 [0.0, 0.0] points) and red meat (0.0 [0.0, 0.0] points), while the highest scoring were dairy (8.9 [4.7, 10.0] points), starchy vegetables (7.7 [1.3, 10.0] points), fruit (6.8 [2.7, 10.0] points) and vegetables (6.4 [4.4, 8.6] points). Usual energy and nutrient intakes are reported in Table 4.

### 3.3. Nutritional Adequacy

Inadequate nutrient intakes were common, with prevalence estimates highest for calcium (70%), magnesium (48%), vitamin E (42%), vitamin B6 (39%), zinc (34%), vitamin A (26%), vitamin B1 (15%), iron (14%) and vitamin B2 (11%). Inadequacy of intake for iodine, vitamin B9, selenium, vitamin C, vitamin B12, B3 and phosphorus was less common, with prevalence below 10% (Table 5).

Higher 2025 PHD adherence was significantly associated with lower odds of inadequate intakes for several micronutrients (Table 5). For each 10-point increase in the PHD adherence score, the odds of inadequacy decreased significantly for vitamins A (OR: 0.803; 95% CI: 0.749, 0.861; *p* < 0.001), B1 (OR: 0.909; 95% CI: 0.837, 0.987; *p* = 0.024), B6 (OR: 0.807; 95% CI: 0.742, 0.878; *p* < 0.001), B9 (OR: 0.870; 95% CI: 0.776, 0.976; *p* = 0.018), C (OR: 0.526; 95% CI: 0.464, 0.595; *p* < 0.001) and E (OR: 0.605; 95% CI: 0.560, 0.653; *p* < 0.001). Protective associations were also observed for magnesium (OR: 0.676; 95% CI: 0.626, 0.730; *p* < 0.001) and selenium (OR: 0.824; 95% CI: 0.745, 0.912; *p* < 0.001). For iron, for each 10-point increase in the PHD adherence score, the probability of inadequate intakes reduced by 1.7 percentage points (coefficient = −0.017; 95% CI: −0.022, −0.011; *p* < 0.001). By contrast, for each 10-point change in the PHD adherence score, the odds of inadequacy increased significantly for both vitamin B12 (OR: 1.239; 95% CI: 1.062, 1.446; *p* = 0.007) and calcium (OR: 1.088; 95% CI: 1.009, 1.173; *p* = 0.029) (Table 5). No significant associations were observed for vitamin B2, iodine and zinc (all *p* > 0.05). The odds for each 1-point increase in adherence score are reported in Supplementary Table S2.

### 3.4. Sociodemographics

All sociodemographic variables were associated with PHD adherence scores in univariable models (all *p* < 0.01) (Table 6). In the multivariable regression model, several sociodemographic factors were independently and positively associated with PHD adherence (Table 7). Higher adherence was observed among older compared to younger individuals (β per each additional year = 0.135; 95% CI: 0.089, 0.180; *p* < 0.001), females compared to males (β = 3.931; 95% CI: 2.579, 5.284; *p* < 0.001), those born in other countries compared to those born in Australia (β = 4.993; 95% CI: 3.398, 6.587; *p* < 0.001) and non-smokers compared to smokers (β = 2.891; 95% CI: 1.500, 4.283; *p* < 0.001). PHD adherence was significantly higher among individuals with higher levels of educational attainment compared to those who did not complete secondary school. The largest differences were observed among those with postgraduate degrees (β = 6.465; 95% CI: 4.521, 8.409; *p* < 0.001) and bachelor’s degrees (β = 4.936; 95% CI: 2.935, 6.936; *p* < 0.001). Additionally, living in a two-person household was associated with greater PHD adherence compared to living alone (β = 1.784; 95% CI: 0.537, 3.032; *p* = 0.006). A non-linear association was observed between alcohol consumption and adherence (*p* < 0.001) characterised by a modest positive trend at 20–30 g/day, with a progressive decline beyond consumption of 40 g/day. In contrast, individuals with obesity had significantly lower PHD adherence scores compared to those with BMIs in the healthy weight range (β = −1.620; 95% CI: −2.828, −0.413; *p* = 0.009) (Table 7). No significant associations were detected between household socioeconomic position (as measured by IRSD or labour force status) and PHD adherence scores (all overall *p*-values > 0.05). Linear regression post-estimation indicated no evidence of problematic multicollinearity, with a mean variance inflation factor of 1.45 (max 1.82).

For sensitivity analyses, we also used the 2019 version of the PHD dietary recommendations, and there were no appreciable changes to any of the associations in the nutrient adequacy and sociodemographic models.

## 4. Discussion

To our knowledge, this is the first study to examine associations between 2025 PHD adherence and nutritional adequacy, as well as sociodemographic factors, in a nationally representative sample of Australian adults. Our findings demonstrate that low overall PHD adherence is driven by underconsumption of wholegrains, legumes, nuts and seeds and overconsumption of added sugars and red meats. Our analyses show that greater PHD adherence may reduce the odds of inadequate intake of several vitamins and minerals while increasing the odds of inadequate vitamin B12 and calcium intake. Our results suggest that PHD adherence may be shaped by a complex interplay of demographic, cultural and behavioural factors, with higher adherence observed among older adults, females, those with higher educational attainment, migrants born in countries where English is not the main language and non-smokers, and lower adherence among those with obesity and those with higher alcohol consumption.

### 4.1. Nutritional Adequacy

Our results largely support the EAT-Lancet Commission’s conclusion that improved PHD adherence can support adequate intake of most nutrients [4], but with important distinctions. Our findings closely mirror those reported across eight Latin American countries, where greater 2019 PHD adherence was associated with increased risks of calcium and vitamin B12 inadequacy, despite reduced risks of deficiency for the other nutrients assessed [13]. Similar trends were reported in the United States, where the probability of inadequate calcium intake appeared to be higher and the probability of inadequate iron lower among individuals in higher 2019 PHD adherence quintiles in a representative sample [9]. Although both studies used methods similar to those employed in the present study, comparisons are limited by differing statistical approaches and by dietary index modifications that are tailored to the available dietary data, which shape how adherence is scored and interpreted [34]. Regardless, there may be trade-offs for a small number of critical nutrients with increasing PHD adherence, unless diets are appropriately optimised.

The prevalence of inadequate calcium intake was high (70%). The higher odds of inadequate intake among those with greater PHD adherence likely reflect reduced dairy consumption, as recommended by the PHD, together with generally low intakes of other calcium-containing foods (wholegrains, legumes, nuts and seeds and fortified plant-based alternatives). In this context, lower dairy consumption is not being offset by increased consumption of these non-dairy calcium sources, resulting in low overall calcium intake [4]. Comparatively, higher odds of inadequate vitamin B12 intake with greater PHD adherence were expected, given the PHD’s emphasis on reducing animal-source foods and our exclusion of supplement users, although the overall prevalence of inadequate B12 intake was low (3%). These findings support the 2025 EAT-Lancet Commission’s acknowledgement that these two nutrients require particular attention in some contexts. Should dietary shifts increasingly align with PHD recommendations, an emphasis on strategic food choices will be important to improve nutritional adequacy. In particular, vitamin B12 supplementation/fortification, alongside calcium fortification, should be prioritised to offset reductions in animal-source foods and dairy [3].

For minerals like iron and zinc, the adequacy of intake is more complex. In our sample, the prevalence of inadequate intake was high for zinc (34%) and moderate for iron (14%). Since total intake is not directly proportional to absorption, these estimates may not reflect the proportion of the nutrient that is bioavailable [35]. For example, vegetarian diets exclude animal tissue and do not contain highly bioavailable haem iron, instead containing only non-haem iron, which is less bioavailable. As a result, iron requirements for vegetarians are up to 80% higher to achieve an equivalent absorption of iron [24]. This reduced iron bioavailability is due to intakes of non-haem iron combined with higher intakes of compounds such as phytates and polyphenols that are found in plant foods, which inhibit iron absorption [35,36]. Zinc absorption is similarly affected by phytates, with requirements up to 50% greater in high-phytate vegetarian diets [32]. Calcium absorption may also be reduced by oxalates found in some plant foods, though the magnitude of this effect and the amount consumed in PHDs remain poorly quantified [37]. Future research should quantify the intake of haem and non-haem iron, zinc and calcium derived from different sources, as well as the presence of known absorption inhibitors in the PHD. Such data could clarify whether the PHD provides sufficient amounts of bioavailable iron, zinc and calcium to meet physiological requirements [38]. Standard assumptions about mineral bioavailability may not apply within this dietary context; without accounting for potential differences in absorption, nutrient adequacy may be overestimated, even when reported intakes appear sufficient.

### 4.2. Demographic Factors

We found several strong associations that contribute to understanding the sociodemographic context of PHDs in Australia. The positive association between age and PHD adherence, also observed elsewhere [9,13,39], is likely explained by overlapping sociodemographic and behavioural mechanisms rather than intentional alignment with healthy or sustainable eating principles. Young adults likely face more barriers to adherence, despite higher motivation, including lower food literacy and cooking skills, financial barriers, more frequent exposure to health and nutrition misinformation online and higher ultra-processed food intakes that displace core PHD foods [40,41,42,43,44,45]. In contrast, older adults may have more stable, traditional or routine eating patterns, which contribute to more home-cooked meals using whole foods, underpinned by differing views about healthy eating compared with young adults [46,47,48,49,50]. Though the effect size for age appears small, we estimate a 35-year age gap to produce a ~4.7-point adherence score increase, equivalent to a ~100 g/day increase in wholegrains and ~50 g/day of fruit (at 2400 kcal/day). This association, however, is best interpreted alongside other correlates of adherence.

Broader attitudes toward PHDs in Australia remain poorly understood [51,52]. Gendered beliefs regarding meat, masculinity and identity may partly explain females’ significantly higher adherence scores, consistent with findings from the United States [9]. Females may face fewer identity and gender-based barriers to reducing meat intake in favour of substitution with plant-based foods, and have elsewhere been shown to exhibit greater pro-environmental values and behaviours than males [53,54]. This is supported by national data showing that females are less likely to consume meat, poultry and game foods [55]. In Australia, a greater proportion of females also report being on a diet, and females are more likely to meet guidelines for fruit and vegetable intake and consume fewer discretionary foods than males [55,56]. The positive association between educational attainment and PHD adherence found in our study aligns with previous research linking higher education to both PHD adherence [9,13,14,57] and overall diet quality [58]. This may reflect a greater awareness of nutrition and sustainability-related issues among individuals with higher education across both sexes.

### 4.3. Intersecting Sociodemographic and Cultural Factors

Although no consistent association was observed between adherence and socioeconomic status, as measured by the IRSD in our study, PHD adherence may not map neatly onto area-level disadvantage. Nonetheless, dietary patterns are shaped by broader structural inequities [59]. Although the PHD may be less expensive than a typical Australian diet [60], recommended diets remain unaffordable for individuals in disadvantaged areas [61]. Moreover, we also observed a negative association between obesity and PHD adherence, consistent with data linking lower diet quality to obesity [62]. Nationally, obesity and smoking prevalence are higher in regional and disadvantaged areas [63], where cumulative disadvantages make highly processed, less sustainable foods more financially accessible, and where fast-food outlets disproportionately concentrate [64,65]. Various structural influences mediate the relationship between disadvantage, dietary behaviours, diet quality and obesity, which merit further investigations to support healthier, more equitable dietary shifts across Australia [66].

We found that only individuals in two-person households (who may include de facto or childless couples and cohabiting adults) had higher PHD adherence scores than those living alone. Shared food purchasing responsibilities, improved meal motivation, companionship, the benefit of dual incomes shared between two people or fewer competing household demands that increase the complexity of the within-house food dynamics may be supportive of healthier food choices in this dynamic [67]. Lastly, higher adherence was found amongst migrants born in countries where English is not the main language, where culturally distinct dietary habits and food traditions, such as vegetarianism, emphasise foods found in the PHD [68]. Shifts toward the PHD should consider the persistence of traditional eating habits and preserve sustainable food practices embedded within migrant or other indigenous food cultures [69].

Our initial framing of Australian PHD adherence in this study highlights the complex web of interconnected sociodemographic factors which influence food literacy, access and affordability as well as dietary habits [70]. Future research should continue to explore these barriers and enablers of PHD transitions—transitions that must also navigate the broader Australian socio-political landscape, influenced by dominant food and industry sectors, cultural norms and competing policy priorities [71,72].

### 4.4. Strengths and Limitations

This research used the most recently available national dataset, a large representative sample, with dietary data collected using a gold-standard method [73,74]. Population-level dietary patterns are unlikely to have shifted substantially since data collection, as demonstrated elsewhere [9]. Our usual dietary intake estimations were conducted using a validated method that accounts for within-person variation [23]. Moreover, the proportional scoring approach used in the HRDS with those intakes has been shown to provide greater precision for epidemiological investigations assessing nutritional adequacy than binary approaches [34]. Our energy-adjustment modification to the HRDS further improved its applicability by avoiding static calorie targets and fixed intake distributions; additional adjustment for energy intake during modelling further controlled for any residual confounding. In addition, our inclusion of numerous sociodemographic variables enabled the identification of equity-relevant disparities in dietary adherence.

Limitations of this study must be noted. The cross-sectional design of this study precludes causal inference between predictors and dietary adherence. Additionally, the updated 2025 PHD included a target for sodium intake (<2 g/day); however, sodium was not incorporated into the 2025 EAT-Lancet Commission’s modelling due to measurement limitations. The same limitation applied to the NNPAS dataset, which did not capture discretionary salt use and therefore underestimated true sodium intake. Accordingly, we excluded sodium from our analyses.

## 5. Conclusions

We contribute a new geographical context to the emerging field of planetary health nutrition which highlights the urgent need to bridge the wide gap between evolving healthy and sustainable dietary recommendations and current consumption patterns in Australia. While greater PHD adherence was generally associated with improvements in achieving nutritional adequacy, specific risks for inadequate intake of calcium and vitamin B12 highlight the importance of sustainability-aligned dietary patterns that emphasise strategic food substitution, fortified foods and possibly supplementation. Observed sociodemographic disparities in adherence further emphasise the need to consider sex, education levels, cultural dietary traditions and broader structural factors in shaping effective interventions for sustainable dietary shifts. As Australia confronts the dual challenge of improving nutritional adequacy and environmental sustainability, targeted, equity-conscious strategies will be critical for reducing nutrition-related health risks while advancing environmental goals.

## Figures and Tables

**Table 1:** Healthy Reference Diet Score design with target values (g/day) based on the 2025 EAT-Lancet Planetary Health Diet of 2400 kcal/day ^1^.

Dietary Components	PHD Intake Target	Proportional Score (0–10)	Max Points (10)	Inverse Score (10–0)
**Adequacy Components**				
Wholegrains	420 g ^2^	0–420 g	≥420 g	
Vegetables	300 g	0–300 g	≥300 g	
Fruits	200 g	0–200 g	≥200 g	
Legumes ^3^	75 g	0–75 g	≥75 g	
**Optimum Components ^4^**				
Starchy Vegetables	50 g	0–50 g	50–100 g	100–150 g
Dairy	250 g	0–250 g	250–500 g	500–750 g
Eggs	15 g	0–15 g	15–25 g	25–40 g
Poultry	30 g	0–30 g	30–60 g	60–90 g
Fish and Seafood	30 g	0–30 g	30–100 g	100–130 g
Nuts and Seeds	50 g	0–50 g	50–75 g	75–125 g
**Moderation Components**				
Red Meats	15 g		0 g	15–0 g
Added Sugars	30 g		0 g	30–0 g
**Ratio Component ^5^**	**15th Percentile**	**85th Percentile**	**Proportional Score** **(0–10)**	**Max Points** **(10)**
Unsaturated/Saturated Fats	1.072	1.829	1.072–1.829	≥1.829

PHD, Planetary Health Diet. ^1^ For individuals in the present study, usual energy intakes were divided by the 2400 kcal/day intake targets to obtain a ratio. Each target was multiplied by this ratio to establish personalised energy-adjusted targets for each dietary component. The ratio component (unsaturated/saturated fats) was excluded from this adjustment. ^2^ The PHD recommendation for wholegrains was converted from dry weight, as described earlier [27]. ^3^ Legumes include fortified soy milks. Only the soy component of the beverage contributed to the estimation of usual intake. ^4^ Maximum point ranges for optimum components are based on the 2025 EAT-Lancet PHD intake ranges [4]. Inverse scores in this system are symmetrical from 0 g up to the lower bound of the maximum point range. ^5^ Due to an absence of disaggregated data for food items such as palm oil, a ratio of unsaturated to saturated fats was used in line with previous research [26,28]. The 15th and 85th percentiles of intake were derived from the present sample using methods described earlier [29].

**Table 2:** Population characteristics and their distribution according to the level of adherence ^1^ to the 2025 EAT-Lancet Planetary Health Diet.

Population Characteristic	Total Sample (*n* = 5655)	Q1 Lowest (6–41) ^2^ (*n* = 1499)	Q2 (42–50) ^2^ (*n* = 1381)	Q3 (51–59) ^2^ (*n* = 1365)	Q4 Highest (60–104) ^2^ (*n* = 1410)
**Age**	**Median (25th, 75th percentile) ^3^**				
Years	43 (29, 57)	40 (26, 53)	42 (28, 57)	43 (30, 57)	47 (33, 59)
**2025 PHD Adherence Score**	**Mean (SE) ^3^**				
Possible range 0–130	50 (0.3)	33 (0.3)	46 (0.1)	55 (0.1)	67 (0.2)
**Sex**	***n*** ** (%) ^3^**	***n*** ** (%) ^3^**	***n*** ** (%) ^3^**	***n*** ** (%) ^3^**	***n*** ** (%) ^3^**
Female	2715 (45%)	604 (37%)	660 (45%)	660 (45%)	791 (55%)
Male	2940 (55%)	895 (63%)	721 (55%)	705 (55%)	619 (45%)
**Body Mass Index (kg/m^2^)**					
Underweight (<18.5)	75 (2%)	21 (2%)	21 (2%)	15 (2%)	18 (2%)
Healthy Weight Range (18.5 to <25)	1602 (35%)	397 (34%)	403 (37%)	374 (34%)	428 (37%)
Overweight (25 to <30)	1703 (35%)	432 (34%)	380 (31%)	432 (38%)	459 (38%)
Obese (≥30)	1362 (27%)	373 (31%)	359 (29%)	337 (26%)	293 (23%)
Missing	913	276	218	207	212
**Education Status**					
Did Not Complete Secondary School	1631 (26%)	504 (31%)	439 (29%)	368 (25%)	320 (21%)
Completed Secondary School	736 (15%)	194 (16%)	175 (15%)	173 (15%)	194 (15%)
Certificate, Trade or Diploma	1922 (35%)	559 (40%)	488 (36%)	436 (32%)	439 (33%)
Bachelor’s Degree	894 (16%)	166 (11%)	195 (15%)	251 (19%)	282 (20%)
Postgraduate Degree	395 (7%)	54 (3%)	71 (5%)	113 (8%)	157 (11%)
Missing or Not Determined	77	22	13	24	18
**Country of Birth**					
Australia	4110 (70%)	1189 (76%)	1023 (72%)	990 (71%)	908 (61%)
Major English-Speaking ^4^	673 (11%)	171 (12%)	167 (12%)	158 (10%)	177 (12%)
Other	872 (19%)	139 (12%)	191 (16%)	217 (19%)	325 (28%)
**Household Size**					
1 Person	1470 (13%)	420 (14%)	351 (12%)	358 (13%)	341 (12%)
2 Persons	1832 (32%)	428 (27%)	443 (31%)	441 (31%)	520 (37%)
3 Persons	917 (20%)	243 (19%)	243 (21%)	237 (21%)	194 (17%)
≥4 Persons	1436 (36%)	408 (39%)	344 (36%)	329 (34%)	355 (34%)
**IRSD ^5^**					
Lowest Quintile (Most Disadvantage)	1155 (20%)	371 (24%)	307 (22%)	242 (17%)	235 (16%)
Second Quintile	1220 (21%)	326 (21%)	332 (23%)	284 (21%)	278 (21%)
Third Quintile	1093 (20%)	275 (20%)	276 (21%)	261 (19%)	281 (21%)
Fourth Quintile	947 (18%)	243 (17%)	210 (16%)	242 (20%)	252 (18%)
Highest Quintile (Least Disadvantage)	1240 (21%)	284 (18%)	256 (18%)	336 (23%)	364 (23%)
**Labour Force Status**					
Employed	3732 (68%)	984 (68%)	888 (67%)	900 (68%)	960 (70%)
Unemployed	147 (3%)	56 (4%)	42 (4%)	25 (3%)	24 (2%)
Not in the Labour Force	1776 (28%)	459 (28%)	451 (29%)	440 (30%)	426 (28%)
**Smoking Status**					
Yes	1295 (21%)	496 (29%)	336 (23%)	260 (17%)	203 (14%)
No	4360 (79%)	1003 (71%)	1045 (77%)	1105 (83%)	1207 (86%)
**Alcohol Consumption ^6^**	**Median (25th, 75th percentile) ^3^**				
Usual Intake (g/day)	3.7 (0.9, 19.9)	3.7 (1.0, 20.7)	3.4 (0.7, 18.1)	3.7 (1.0, 20.4)	3.6 (0.7, 20.4)

PHD, Planetary Health Diet; IRSD, Index of Relative Socio-economic Disadvantage. ^1^ Planetary Health Diet adherence was measured using the Healthy Reference Diet Score [26]. Possible scores range from 0 (lowest) to 130 (highest) points; quartiles (Q1 Lowest, Q4 Highest) were derived from the observed score range (6–104 points). ^2^ A range (min–max) of PHD adherence scores per quartile. ^3^ Mean (SE) and median (25th, 75th percentile) were calculated using the survey sampling weights to account for the complex survey design; *n* (% = survey-weighted proportions); values may not sum to 100% due to rounding. ^4^ Major English-speaking countries include Canada, Ireland, New Zealand, South Africa, the United Kingdom and the United States of America, as specified in the Standard Australian Classification of Countries [20]. ^5^ The Index of Relative Socio-economic Disadvantage reflects the relative level of disadvantage in an area based on variables such as income, education level, unemployment and occupation. Lower quintiles indicate greater disadvantage [20]. ^6^ 10 g of ethanol is equivalent to one standard drink [31]. Usual intakes of alcohol were estimated using the Multiple Source Method [23].

**Table 3:** Median ^1^ (25th, 75th percentile) usual dietary component intakes (g/day) ^2^ and their distribution according to the level of adherence ^3^ to the 2025 EAT-Lancet Planetary Health Diet.

Dietary Components	Usual Population Intake	Q1 (Lowest) (6–41) ^4^ *n* = 1530	Q2 (42–50) ^4^ *n* = 1302	Q3 (51–59) ^4^ *n* = 1412	Q4 (Highest) (60–104) ^4^ *n* = 1411	Median Component Scores ^5^ (0–10)
**Adequacy Components**	**Median (25th, 75th Percentile)**					
Wholegrains	14.8 (0.0, 48.2)	11.3 (0.0, 36.9)	13.4 (0.0, 41.8)	16.9 (6.1, 52.7)	28.1 (8.0, 60.6)	0.4 (0.0, 1.4)
Vegetables	157.1 (111.3, 210.9)	129.6 (86.8, 175.1)	151.1 (105.3, 203.0)	166.1 (123.2, 222.4)	183.7 (139.9, 233.9)	6.4 (4.4, 8.6)
Fruits	109.6 (44.9, 202.4)	49.9 (25.6, 112.2)	92.3 (41.6, 178.0)	128.4 (59.4, 212.6)	183.6 (109.4, 264.3)	6.8 (2.7, 10.0)
Legumes	3.6 (0.0, 11.6)	0.0 (0.0, 6.6)	2.8 (0.0, 9.2)	4.3 (0.0, 12.6)	7.6 (0.0, 29.6)	0.6 (0.0, 2.0)
**Optimum Components**						
Starchy Vegetables	60.6 (32.5, 97.0)	64.4 (20.7, 118.6)	66.5 (33.5, 101.5)	58.3 (34.8, 87.8)	56.9 (36.9, 79.5)	7.7 (1.3, 10.0)
Dairy	308.2 (191.0, 454.1)	365.7 (194.6, 552.0)	321.9 (199.3, 464.8)	292.5 (183.6, 428.8)	273.4 (185.9, 383.7)	8.9 (4.7, 10.0)
Eggs	12.5 (5.4, 24.0)	10.1 (0.0, 24.0)	11.7 (5.3, 24.7)	13.6 (6.9, 24.8)	13.3 (8.5, 22.8)	5.5 (0.0, 9.4)
Poultry	46.4 (18.7, 73.0)	47.2 (0.0, 82.4)	47.7 (16.2, 76.8)	46.9 (25.4, 70.5)	43.0 (27.5, 64.9)	1.3 (0.0, 9.8)
Fish and Seafood	15.3 (0.0, 34.0)	0.0 (0.0, 15.3)	13.9 (0.0, 25.9)	17.4 (4.9, 36.8)	29.3 (14.0, 53.7)	5.6 (0.0, 10.0)
Nuts and Seeds	2.7 (0.0, 7.3)	1.0 (0.0, 3.9)	2.2 (0.0, 5.5)	3.3 (0.0, 8.7)	5.5 (0.8, 14.7)	0.6 (0.0, 1.7)
**Moderation Components**						
Red Meats	82.0 (51.3, 115.2)	89.2 (60.4, 120.1)	83.8 (56.8, 117.5)	85.4 (53.0, 118.4)	69.3 (31.6, 103.4)	0.0 (0.0, 0.0)
Added Sugars	42.1 (24.8, 69.1)	52.3 (29.1, 80.4)	44.2 (26.8, 75.3)	41.0 (24.4, 65.1)	34.2 (20.3, 52.5)	0.0 (0.0, 0.0)
**Ratio Component ^6^**						
Unsaturated Fats	36.4 (28.5, 45.6)	34.7 (26.8, 43.5)	36.3 (27.7, 46.4)	37.0 (29.2, 46.3)	37.3 (30.0, 46.6)	4.1 (1.3, 8.0)
Saturated Fats	26.2 (19.7, 33.5)	28.8 (22.0, 36.9)	27.1 (20.7, 34.8)	25.4 (19.3, 32.7)	23.0 (18.0, 29.5)	

PHD, Planetary Health Diet. ^1^ Survey-weighted medians (25th, 75th percentile) were calculated using the survey sampling weights to account for the complex survey design. ^2^ Usual food group intakes were estimated using the Multiple Source Method [23]. ^3^ Planetary Health Diet adherence was measured using the Healthy Reference Diet Score [26]. Possible scores ranged from 0 points (lowest) to a theoretical maximum of 130 points (highest); quartiles (Q1–Q4) were derived from the observed score range (6–104 points). ^4^ A range (min–max) of the PHD adherence scores per quartile. ^5^ Median (25th, 75th percentile) dietary component scores reflect individual contributions made by each dietary component towards the overall PHD adherence scores. ^6^ The ratio component uses the 15th and 85th percentiles of intake (sample-derived) to establish cut-offs for proportional scoring, using the methods described earlier [29].

**Table 4:** Usual ^1^ daily population nutrient intakes ^2^ and their distribution according to the level of adherence ^3^ to the 2025 EAT-Lancet Planetary Health Diet.

		Usual Population Intake	Q1 (Lowest) (6–41) ^4^ *n* = 1530	Q2 (42–50) ^4^ *n* = 1302	Q3 (51–59) ^4^ *n* = 1412	Q4 (Highest) (60–104) ^4^ *n* = 1411
Nutrient	Units	Mean (SE)				
Energy	kcal/day	2037.2 (8.5)	2063.4 (20.6)	2060.9 (20.2)	2051.1 (19.4)	1973.1 (17.9)
Fibre	g/day	21.7 (0.1)	18.9 (0.3)	21.1 (0.2)	22.6 (0.3)	24.5 (0.3)
Vitamin B1	mg/day	1.5 (0.0)	1.5 (0.0)	1.5 (0.0)	1.5 (0.0)	1.5 (0.0)
Vitamin B2	mg/day	1.8 (0.0)	1.9 (0.0)	1.9 (0.0)	1.8 (0.0)	1.7 (0.0)
Vitamin B3	mg/day	41.3 (0.2)	42.0 (0.4)	41.9 (0.5)	41.1 (0.4)	40.1 (0.4)
Vitamin B6	mg/day	1.5 (0.0)	1.4 (0.0)	1.5 (0.0)	1.5 (0.0)	1.5 (0.0)
Vitamin B9	µg/day	606.2 (4.1)	605.2 (10.3)	605.6 (7.6)	608.1 (8.1)	606.2 (6.4)
Vitamin B12	µg/day	4.5 (0.0)	4.7 (0.1)	4.6 (0.1)	4.4 (0.1)	4.1 (0.1)
Vitamin A	µg/day	790.5 (8.8)	747.1 (12.7)	797.8 (23.8)	802.8 (16.0)	817.2 (10.9)
Vitamin C	mg/day	98.5 (1.1)	83.3 (1.7)	94.4 (2.4)	104.2 (2.7)	113.4 (2.3)
Vitamin E	mg/day	9.8 (0.1)	8.6 (0.1)	9.6 (0.1)	10.1 (0.1)	11.0 (0.1)
Calcium	mg/day	776.3 (5.3)	816.6 (13.2)	779.5 (11.3)	766.7 (9.9)	739.9 (8.0)
Iodine	µg/day	170.4 (0.9)	178.1 (2.2)	173.4 (2.1)	168.6 (2.1)	161.1 (1.7)
Iron	mg/day	10.7 (0.1)	10.3 (0.1)	10.7 (0.1)	10.9 (0.1)	11.1 (0.1)
Magnesium	mg/day	321.0 (1.7)	302.5 (3.6)	313.2 (3.2)	329.7 (3.2)	340.0 (3.5)
Phosphorus	mg/day	1430.4 (5.9)	1448.8 (13.8)	1441.1 (14.1)	1437.1 (12.9)	1393.9 (13.9)
Selenium	µg/day	88.1 (0.4)	84.9 (1.0)	87.3 (1.0)	90.1 (1.1)	90.3 (1.2)
Zinc	mg/day	10.7 (0.1)	10.8 (0.1)	10.8 (0.1)	10.9 (0.1)	10.4 (0.1)

PHD, Planetary Health Diet. ^1^ Survey-weighted means and standard errors were calculated using the survey sampling weights to account for the complex survey design. ^2^ Usual nutrient intakes were estimated using the Multiple Source Method [23]. ^3^ Planetary Health Diet (PHD) adherence was measured using the Healthy Reference Diet Score [26]. Possible scores range from 0 points (lowest) to a theoretical maximum of 130 points (highest); quartiles were derived from the observed score range (6–104 points). ^4^ A range (min–max) of the PHD adherence scores per quartile.

**Table 5:** Prevalence of inadequate nutrient intake and odds ^1^ of inadequate nutrient intake per 10-point increase in adherence to 2025 EAT-Lancet Planetary Health Diet.

Nutrient	Adequacy Category ^2^	*n* (%) ^3^	Odds Ratio	95% CI	*p*-Value
Vitamin A	Adequate (reference)	4204 (74%)	1		
	Inadequate	1451 (26%)	0.803	0.749, 0.861	*p* < 0.001
Vitamin B1	Adequate (reference)	4803 (85%)	1		
	Inadequate	852 (15%)	0.909	0.837, 0.987	0.024
Vitamin B2	Adequate (reference)	5009 (89%)	1		
	Inadequate	646 (11%)	0.915	0.830, 1.010	0.076
Vitamin B6	Adequate (reference)	3423 (61%)	1		
	Inadequate	2232 (39%)	0.807	0.742, 0.878	*p* < 0.001
Vitamin B9	Adequate (reference)	5281 (93%)	1		
	Inadequate	374 (7%)	0.870	0.776, 0.976	0.018
Vitamin B12	Adequate (reference)	5474 (97%)	1		
	Inadequate	181 (3%)	1.239	1.062, 1.446	0.007
Vitamin C	Adequate (reference)	5307 (94%)	1		
	Inadequate	348 (6%)	0.526	0.464, 0.595	*p* < 0.001
Vitamin E	Adequate (reference)	3269 (58%)	1		
	Inadequate	2386 (42%)	0.605	0.560, 0.653	*p* < 0.001
Calcium	Adequate (reference)	1715 (30%)	1		
	Inadequate	3940 (70%)	1.088	1.009, 1.173	0.029
Iodine	Adequate (reference)	5231 (93%) ^4^	1		
	Inadequate	424 (8%) ^4^	0.978	0.880, 1.088	0.680
Magnesium	Adequate (reference)	2962 (52%)	1		
	Inadequate	2693 (48%)	0.676	0.626, 0.730	*p* < 0.001
Selenium	Adequate (reference)	5253 (93%)	1		
	Inadequate	402 (7%)	0.824	0.745, 0.912	*p* < 0.001
Zinc	Adequate (reference)	3722 (66%)	1		
	Inadequate	1933 (34%)	0.950	0.884, 1.020	0.151
Iron ^5^	% of inadequate intakes	14%	**Coefficient**	**95% CI**	***p*** **-value**
			−0.017	−0.022, −0.011	*p* < 0.001

^1^ The logistic regression models were each survey-weighted and adjusted for age, sex and usual energy intake. ^2^ Nutritional adequacy was determined using usual nutrient intakes and the age- and sex-specific Estimated Average Requirement Cut-Point Method as per the Australian/New Zealand Nutrient Reference Values for all nutrients except iron, for which the full probability method was used [24]. ^3^ Proportions reflect survey-weighted estimates. ^4^ Values that exceed 100% are due to rounding. ^5^ For iron, a linear regression was used to model the probability of inadequate intakes, as is necessary when using the full probability method [32].

**Table 6:** Univariable regression ^1^ assessing associations between adherence to the 2025 EAT-Lancet Planetary Health Diet Scores and sociodemographic factors.

Population Characteristic	Coefficient	95% CI	*p*-Value ^2^
**Age**	0.090	0.059, 0.121	*p* < 0.001
**Sex**			*p* < 0.001
*Male*			
Female	3.331	2.042, 4.621	
**BMI** (kg/m^2^)			0.005
*Normal (18.5 to <25)*			
Underweight (<18.5)	0.738	−4.770, 6.245	
Overweight (25 to <30)	0.560	−0.713, 1.832	
Obese (≥30)	−2.013	−3.238, −0.787	
**Education**			*p* < 0.001
*Did Not Complete Secondary School*			
Completed Secondary School	1.784	0.148, 3.419	
Certificate, Trade or Diploma	0.278	−1.016, 1.573	
Bachelor’s Degree	5.379	3.489, 7.268	
Postgraduate Degree	7.983	5.987, 9.980	
**Country of Birth**			*p* < 0.001
*Australia*			
Major English-Speaking ^3^	0.818	−1.132, 2.768	
Other	5.605	4.073, 7.137	
**Household Size**			0.008
*1 person*			
2 persons	2.044	0.761, 3.326	
3 persons	0.371	−1.215, 1.958	
≥4 persons	0.253	−1.005, 1.511	
**IRSD**			0.002
*Lowest Quintile*			
Second Quintile	1.665	0.261, 3.068	
Third Quintile	2.231	0.479, 3.982	
Fourth Quintile	2.473	0.798, 4.149	
Fifth Quintile	3.514	1.779, 5.249	
**Labour Force Status**			0.009
*Employed*			
Unemployed	−4.658	−7.583, −1.733	
Not in the labour force	0.461	−0.762, 1.685	
**Smoking Status**			*p* < 0.001
*Yes*			
No	4.798	3.436, 6.160	
**Alcohol Consumption ^4^**			*p* < 0.001
Usual Intakes (g/day)			

IRSD, Index of Relative Socio-economic Disadvantage. ^1^ Linear regression on complete cases (*n* = 4678) excluded 977 adults missing BMI or education and was adjusted for energy intake. ^2^ Overall *p*-values are derived from the adjusted Wald’s Test assessing the joint significance of all categories within each categorical variable. Continuous and binary variables are not included in these tests. ^3^ Major English-speaking countries include Canada, Ireland, New Zealand, South Africa, the United Kingdom and the United States of America, as specified in the Standard Australian Classification of Countries [33]. ^4^ Alcohol consumption was modelled using restricted cubic splines to account for non-linearity. The reported *p*-value reflects the joint significance of all spline terms from Wald’s test.

**Table 7:** Multivariable Regression ^1^ assessing independent associations between adherence to the 2025 EAT-Lancet Planetary Health Diet Scores and sociodemographic factors.

Population Characteristic	Coefficient	95% CI	*p*-Value	Overall *p*-Value ^2^
**Age**	0.135	0.089, 0.180	*p* < 0.001	
**Sex**				
*Male*				
Female	3.931	2.579, 5.284	*p* < 0.001	
**BMI (kg/m^2^)**				
*Healthy Weight Range (18.5 to <25)*				
Underweight (<18.5)	2.342	−2.693, 7.378	0.356	0.018
Overweight (25 to <30)	0.473	−0.709, 1.655	0.427	
Obese (≥30)	−1.620	−2.828, −0.413	0.009	
**Education**				
*Did Not Complete Secondary School (<Year 12)*				
Completed Secondary School (Year 12)	3.060	1.397, 4.722	0.001	*p* < 0.001
Certificate, Trade or Diploma	1.432	0.142, 2.723	0.030	
Bachelor’s Degree	4.936	2.935, 6.936	*p* < 0.001	
Postgraduate Degree	6.465	4.521, 8.409	*p* < 0.001	
**Country of Birth**				
*Australia*				
Major English-Speaking **^3^**	−0.060	−1.879, 1.758	0.947	*p* < 0.001
Other	4.993	3.398, 6.587	*p* < 0.001	
**Household Size**				
*1 person*				
2 persons	1.784	0.537, 3.032	0.006	0.029
3 persons	0.801	−0.720, 2.322	0.296	
≥4 persons	0.397	−0.907, 1.701	0.545	
**IRSD**				
*Lowest Quintile–Most Disadvantaged*				
Second Quintile	1.155	0.011, 2.298	0.048	0.219
Third Quintile	1.169	−0.459, 2.798	0.156	
Fourth Quintile	1.223	−0.309, 2.756	0.115	
Fifth Quintile–Least Disadvantaged	1.414	−0.040, 2.868	0.056	
**Labour Force Status**				
*Employed*				
Unemployed	−2.567	−5.789, 0.654	0.116	0.178
Not in the Labour Force	−0.721	−2.278, 0.835	0.357	
**Smoking Status**				
*Yes*				
No	2.891	1.500, 4.283	*p* < 0.001	
**Alcohol Consumption**				
Usual Intakes (g/day) ^4^				*p* < 0.001

IRSD, Index of Relative Socio-economic Disadvantage. ^1^ Linear regression on complete cases (*n* = 4678) excluded 977 adults missing BMI or education and was adjusted for energy intake. ^2^ The overall *p*-values are derived from the adjusted Wald’s Test assessing the joint significance of all categories within each categorical variable. Continuous and binary variables are not included in these tests. ^3^ Major English-speaking countries include Canada, Ireland, New Zealand, South Africa, the United Kingdom and the United States of America as specified in the Standard Australian Classification of Countries [20]. ^4^ Alcohol consumption was modelled using restricted cubic splines to account for non-linearity. The reported *p*-value reflects the joint significance of all spline terms from Wald’s test.

## Data Availability

Data described in the manuscript, code book, and analytic code may be made available upon request pending application to and approval by the Australian Bureau of Statistics and payment of any necessary data fees: https://www.abs.gov.au/statistics/microdata-tablebuilder/available-microdata-tablebuilder/australian-health-survey-nutrition-and-physical-activity (accessed on 3 November 2025).

## Supplementary Materials

The following supporting information can be downloaded at https://www.mdpi.com/article/10.3390/nu18020340/s1, Table S1. Healthy Reference Diet Score Food Group Inclusions and Exclusions. Foods Matched and Included According to the Available NNPAS Data in Alignment with the 2025 EAT-Lancet Planetary Health Dietary Recommendations. Table S2. Prevalence of Inadequate Nutrient Intake and Odds of Inadequate Nutrient Intake per 1-point Increase in Adherence to the 2025 EAT-Lancet Planetary Health Diet.
