# Supplementary material for: Associations Between Adherence to the EAT-Lancet Planetary Health Diet and Nutritional Adequacy, and Sociodemographic Factors Among Australian Adults
Source: Nutrients. 2026 Jan 21;18(2):340. doi: 10.3390/nu18020340 (PMC12844771; doi:10.3390/nu18020340)
Supplement: Supplementary file 1 [file nutrients-18-00340-s001.zip › nutrients-4064761-supplementary.pdf]

Table S1. Healthy Reference Diet Score Food Group Inclusions and Exclusions. Foods Matched and Included According to the Available NNPAS Data in Alignment with the 2025 EAT-Lancet Planetary Health Dietary Recommendations.

Dietary Components	Inclusions	Exclusions
**Adequacy Components**		
**Wholegrains**	• Wholemeal or mixed grain breads, flatbreads, and rolls<br>• Cooked brown rice, wholemeal pasta, buckwheat and soba noodles, barley, bulgur, sorghum, quinoa, and popcorn<br>• Cooked whole oats (traditional and quick varieties), mixed grain cereal flakes, and muesli<br>• Savoury crackers or crispbreads made from wholemeal wheat flour or rye flour<br>• Wholemeal crumpets, wholemeal or buckwheat crepes, pikelets, and pancakes<br>• Wholemeal or mixed grain English muffins and scones<br>• Flours: wholemeal wheat flour, wholemeal rye flour, barley flour, buckwheat flour, oat flour, spelt flour, soy flour, and chickpea flour	• White wheat flour breads, flatbreads, and rolls<br>• Cooked white rice, white wheat flour pasta, noodles, semolina, polenta, couscous, sago, and tapioca<br>• Cornflakes, puffed corn, rice and oat flakes<br>• Savoury crackers or crispbreads made from white wheat flour, rice flour, or corn<br>• White wheat flour crumpets, crepes, pikelets, and pancakes<br>• White wheat flour English muffins and scones<br>• White wheat flour, rice flour, arrowroot flour, and cornflour
**Vegetables**	• Green and brassica vegetables: Broccoli, broccolini, Chinese broccoli, Brussels sprouts, cabbage, Chinese cabbage, cauliflower, kale, peas, beans, asparagus, snow peas, lettuce, silver beet, spinach, rocket, watercress, chicory, and fresh herbs<br>• Root vegetables: Carrot and pumpkin<br>• Other vegetables: Beetroot, celeriac, radish, swede, turnip, bamboo shoots, celery, sprouts, tomato, squash, zucchini, avocado, capsicum, chilli, choko, cucumber, eggplant, okra, artichoke, mushroom, onion/shallots, ginger, garlic, fennel, leek, seaweed, olives, capers, and horseradish	• Starchy vegetables (categorised elsewhere)<br>• Vegetable juices
**Fruit**	• Fresh, frozen, and canned fruits: Whole, sliced, or diced fresh fruit; frozen fruit; and canned fruit<br>• Dried fruits: Dried apricots, dried fruit salad, dried dates, sultanas, and dried berries	• Fruit juices (100% juice), cordials, fruit drinks (less than 100% juice), confectionary with added juice<br>• Jams
**Legumes[1]**	• Baked beans, canned beans, cooked dried beans, lentils, chickpeas, split peas<br>• Vegetable patties (legume-based, typically lentil or chickpea)<br>• Tofu, tempeh<br>• Soy-based vegetarian sausages and meat<br>• Soy milks (includes fortified), soy yoghurts and soy cheeses	• Legume-based protein powders.

**Optimum Components**		
**Starchy Vegetables**	• Potato, white & orange sweet potato, taro, cassava, sweetcorn, parsnip	
**Dairy**	• All dairy milks, yoghurts, cheeses and creams	• Butters and butter blend spreads (including ghee)<br>• Alternative milks (soy, almond, rice, oat)<br>• Dairy protein powders.
**Poultry**	**Poultry, lean (<10% fat):**<br>• Unprocessed poultry (<10% fat): Cooked chicken, turkey, goose, duck, and quail with fat content <10%<br>• Processed poultry (<10% fat): Roast turkey deli slice, processed turkey luncheon meat, low- or reduced-fat processed chicken luncheon meat, and canned chicken breast in brine<br><br>**Poultry, non-lean (≥10% fat):**<br>• Unprocessed poultry (≥10% fat): chicken, turkey, goose, duck, quail, squab, and emu with fat content ≥10%<br>• Processed poultry (≥10% fat): includes regular-fat processed chicken luncheon meat	
**Eggs**	• Whole eggs, egg yolks, egg whites	
**Fish and Seafood**	• Includes both fresh and canned fish, prawns, crab, lobster, mussels, oysters, scallops and clams	
**Nuts and Seeds**	• Nuts, seeds, nut butters, tahini, nut/seed pastes, no added salt	
**Moderation Components**		

**Red Meat**	**Red meat, lean (<10% fat):**<br>• Unprocessed meat (<10% fat): Cooked lean red meats such as beef, lamb, veal, pork, goat, rabbit, venison, kangaroo, and lean (lower salt) sausages with a fat content <10%<br>• Processed meat (<10% fat): Lean leg ham, trimmed bacon, and lean corned beef<br><br>**Red meat, non-lean (≥10% fat):**<br>• Unprocessed meat (≥10% fat): Cooked beef, lamb, mutton, and pork with fat content ≥10%<br>• Processed meat (≥10% fat): Regular sausages, and non-lean processed meats such as untrimmed bacon, prosciutto, pastrami, salami, and chorizo	
**Added Sugars**	**FSANZ Classification:**<br>• Includes sugars added during manufacturing, preparation, or cooking. Specifically captures table sugars (white, brown, raw, icing sugar), sugar syrups (e.g., glucose syrup, maple syrup, corn syrup, agave syrup, molasses), fruit sugar syrups, and sugar-based confectionery without additions (e.g., plain boiled lollies). Also includes sugars added to foods such as sweetened beverages (e.g., soft drinks, cordials without fruit juice), desserts, baked goods, and breakfast cereals where sugar is an added ingredient.	• Excludes intrinsic sugars from whole fruits, vegetables, and dairy, as well as naturally occurring sugars in honey and 100% fruit juices
**Ratio Component**		
**Saturated Fats**	• Total saturated fat intake from food (g/day)	• SFA supplements
**Unsaturated Fats**	• Total monounsaturated and polyunsaturated fat intake from food (g/day)	• MUFA and PUFA supplements
**No Classification / Excluded from Index Scoring**		
• Bicarbonate soda, cream of tartar, baking powder, gelatine, stock cubes, liquid stock, gravy, salt, dried herbs & spices, curry powder, vinegar, liquid-based sauces/marinades (e.g. soy, fish, teriyaki), yeast & yeast spread, infant formula, human milk, protein powder, alcohol, tea leaves & coffee powder & beans.<br>• White and refined breads/flours as described in the Wholegrains adequacy category above (as per the EAT-Lancet Planetary Health Diet recommendations).		

NNPAS, National Nutrition and Physical Activity Survey. **FSANZ**, Food Standards Australia and New Zealand. **SFA**, Saturated fatty acid. **MUFA**, Monounsaturated fatty acid. **PUFA**, Polyunsaturated Fatty Acid.

Table S2. Prevalence of Inadequate Nutrient Intake and Odds[1] of Inadequate Nutrient Intake per 1-point Increase in Adherence to the 2025 EAT-Lancet Planetary Health Diet.

Nutrient	Adequacy Category[2]	n (%)[3]	Odds Ratio	95% CI	p-value
Vitamin A	Adequate **(reference)**	4,204 (74%)	1		
	Inadequate	1,451 (26%)	0.978	0.972, 0.985	p <0.001
Vitamin B1	Adequate **(reference)**	4,803 (85%)	1		
	Inadequate	852 (15%)	0.990	0.982, 0.999	0.024
Vitamin B2	Adequate **(reference)**	5,009 (89%)	1		
	Inadequate	646 (11%)	0.991	0.982, 1.001	0.076
Vitamin B6	Adequate **(reference)**	3,423 (61%)	1		
	Inadequate	2,232 (39%)	0.979	0.971, 0.987	p <0.001
Vitamin B9	Adequate **(reference)**	5,281 (93%)	1		
	Inadequate	374 (7%)	0.986	0.974, 0.998	0.018
Vitamin B12	Adequate **(reference)**	5,474 (97%)	1		
	Inadequate	181 (3%)	1.022	1.006, 1.038	0.007
Vitamin C	Adequate **(reference)**	5,307 (94%)	1		
	Inadequate	348 (6%)	0.938	0.926, 0.949	p <0.001
Vitamin E	Adequate **(reference)**	3,269 (58%)	1		
	Inadequate	2,386 (42%)	0.951	0.944, 0.958	p <0.001
Calcium	Adequate **(reference)**	1,715 (30%)	1		
	Inadequate	3,940 (70%)	1.008	1.000, 1.016	0.029
Iodine	Adequate **(reference)**	5,231 (93%)[4]	1		
	Inadequate	424 (8%)[4]	0.998	0.987, 1.008	0.680
Magnesium	Adequate **(reference)**	2,962 (52%)	1		
	Inadequate	2,693 (48%)	0.962	0.954, 0.969	p <0.001
Selenium	Adequate **(reference)**	5,253 (93%)	1		
	Inadequate	402 (7%)	0.981	0.971, 0.991	p <0.001
Zinc	Adequate **(reference)**	3,722 (66%)	1		
	Inadequate	1,933 (34%)	0.995	0.988, 1.002	0.151
Iron[5]	% of inadequate intakes	14%	**Coefficient**	**95% CI**	**p-value**
			–0.002	–0.002, –0.001	p <0.001

[1] The logistic regression models were each survey-weighted and adjusted for age, sex and usual energy intake.

[2] Nutritional adequacy was determined using usual nutrient intakes and the age- and sex-specific Estimated Average Requirement-Cut Point method as per the Australian/New Zealand Nutrient Reference Values [1].

[3] Proportions reflect survey-weighted estimates.

[4] Values that exceed 100% are due to rounding.

[5] For iron, a linear regression, adjusted for age sex and energy intake, was used to model the probability of inadequate intakes, as is necessary when using the full probability method [2].

References

1. National Health and Medical Research Council. Nutrient Reference Values: Australia and New Zealand: Australian Government Department of Health and Ageing, New Zealand Ministry of Health; 2006 [cited 2025 May 29]. Available from: https://www.eatforhealth.gov.au/nutrient-reference-values.

2. Institute of Medicine (US). Panel on Micronutrients. Dietary Reference Intakes for Vitamin A, Vitamin K, Arsenic, Boron, Chromium, Copper, Iodine, Iron, Manganese, Molybdenum, Nickel, Silicon, Vanadium, and Zinc. Washington DC: National Academies Press; 2001.
